# Effect of Chronic Morphine Consumption on Synaptic Plasticity of Rat's Hippocampus: A Transmission Electron Microscopy Study

**DOI:** 10.1155/2013/290414

**Published:** 2013-11-28

**Authors:** Mohammad Hassan Heidari, Abdollah Amini, Zohreh Bahrami, Ali Shahriari, Abolfazle Movafag, Reihane Heidari

**Affiliations:** ^1^Department of Biology and Anatomical Sciences, Faculty of Medicine, Shahid Beheshti University of Medical Sciences, Tehran, Iran; ^2^Department of Anesthesiology, Roozbeh Hospital, Tehran University of Medical Sciences, P.O. Box 1417653761, Tehran, Iran; ^3^Department of Genetic Science, Medical Faculty, Shahid Beheshti University of Medical Sciences, Tehran, Iran

## Abstract

It is well known that the synapses undergo some changes in the brain during the course of normal life and under certain pathological or experimental circumstances. One of the main goals of numerous researchers has been to find the reasons for these structural changes. In the present study, we investigated the effects of chronic morphine consumption on synaptic plasticity, postsynaptic density thickness, and synaptic curvatures of hippocampus CA1 area of rats. So for reaching these goals, 24 *N*-Mary male rats were randomly divided into three groups, morphine (*n* = 8), placebo (*n* = 8), and control (*n* = 8) groups. In the morphine group, complex of morphine (0.1, 0.2, 0.3, and 0.4) mg/mL and in the placebo (sucrose) group complex of sucrose (% 0.3) were used for 21 days. After the end of drug treatment the animals were scarified and perfused intracardinally and finally the CA1 hippocampal samples were taken for ultrastructural studies, and then the obtained data were analyzed by SPSS and one-way analysis of variance. Our data indicated that synaptic numbers per nm^3^ change significantly in morphine group compared to the other two groups (placebo and control) (*P* < 0.001) and also statistical analysis revealed a significant difference between groups in terms of thickness of postsynaptic density (*P* < 0.001) and synaptic curvature (*P* < 0.007). It seems that morphine dependence in rats plays a main role in the ultrastructural changes of hippocampus.

## 1. Introduction

Today the opiates are the most addictive substance used in the world wide and they are considered as one of the greatest neuropsychological disorder reasons [1–4]. It has been shown that chronic consumption of opiates can significantly change the functions, structures, and morphology of neural systems [5, 6]. This kind of changes causes several different types of disorders such as drug addiction, carelessness, fluent speech disorders, memory and learning impairment, and psychological disorders [7–10].

According to the studies, drug addiction is an abnormal form of learning and an adaptation of the memory system in certain regions of the brain, such as the hippocampus [11]. The learning processes are defined as a modification in some synaptic functions (synaptic plasticity), changes in the pre- and postsynaptic ultrastructure, and the formation of new synapses [10, 12]. It is known that the synapses of hippocampus formation are more structurally affected by learning process [12–16]. There are previous researchers demonstrate that opiates have either direct or indirect effect on capacity of learning and memory, processes. Concerning the role of the hippocampal synapses in learning, memory, and circuit reward process, it was concluded that morphine consumption as a main opiate substance can cause some functional, structural, and morphological changes in synapses of the hippocampus in the mammalians [17, 18].

However, many works support the link between synaptic plasticity changes and repeated morphine consumptions but only a few studies have recently start to examine the ultrastructural effects of morphine on synapses to explain the relationship between altered connectivity and opiate treatment [4, 19]. So to look into these issues with more details, the current experimental study employed transmission electron microscopy to compare the effects of morphine consumption on synaptic plasticity, postsynaptic density thickness, and synaptic curvatures in CA1 area of hippocampus formations.

The electron microscopy studies could provide some important evidence of synaptic plasticity and the precise pattern of synaptic restructuring.

## 2. Method

### 2.1. Experimental Animals

24 *N*-Mary male rats, weighing 290–300 g and 8–9 weeks of age, were used in this study. They were housed 4 per cage in a climate-controlled room under a 12-hour alternating light/dark cycle at a controlled temperature of 23 ± 1. Dry food pellets and water were provided ad libitum.

### 2.2. Drug

Morphine was purchased from the Darou Pakhsh Pharma Chem company in powder form. Then it was dissolved in tap water to produce solutions of 0.1, 0.2, 0.3, and 0.4 mg/mL (w/v). We dissolved 3 gram of sucrose per 100 mL of solution to reach a sweet solution.

### 2.3. Treatment

After 2 weeks of acclimation to the diet and the environment, 24 *N*-Mary male rats were randomly divided into 3 groups, morphine (*n* = 8), placebo (sucrose) (*n* = 8), and control (water) (*n* = 8) groups. The rats in the morphine groups chronically consumed morphine solutions at doses of 0.1, 0.2, and 0.3 mg/mL for 48 hrs and 0.4 mg/mL up to 21 days. Sucrose (3 g/100 mL) was added to drinking water to mask the bitter taste of morphine. In the placebo group sucrose (3 g per 100 mL) was administrated in drinking water for the same duration of time [10]. In the pilot study, the average water consumption during the administration of the highest dose (0.4 mg/mL) was 50 mg·kg^−1^·day [20, 21].

### 2.4. Electron Microscopy Tissue Preparation

At the end of each drug treatment, all of the rats were deeply anesthetized with 100 mg/kg of pentobarbital and perfused transcardially with 4% paraformaldehyde/0.6% glutaraldehyde in 0.1 M phosphate, pH 7.4. Then, their brains were separated by usual dissection methods and postfixed in 4% paraformaldehyde for 2 h. After fixation, the samples were washed with PBS, then postfixed with 1% osmium tetroxide for 1.5 h, again washed in PBS, dehydrated in an acetone series, and then embedded in epoxy resin [22–24].

For ultrathin sections the block face was first trimmed and the semithin sections were provided at 500 nm thicknesses, stained with 1% toluidine blue, and were examined by light microscopy, for finding the desired area. After finding desired area ultrathin sections (~70 nm-thick) were cut by ultramicrotome (ultracut UCT) with glass knives (Leica EMKMR2) at a thickness of silver-gold interface color and picked up on 100 mesh copper grids. The sections were stained with a 2% aqueous solution of uranyl acetate for 3 min and then with a lead citrate (0.5%) solution for 5–10 min. Ultimately, the samples were viewed on transmission electron microscope (Zeiss EM900) and micrographs were taken at 30,000x magnification. We tried to obtain uniform section thickness at the time of cutting.

### 2.5. Synapse Density

Synapse density was estimated by the dissector method. Eight electron micrographs were taken from three separate regions of each brain (24 micrographs per brain) at a magnification of 30,000x on a Zeiss EM900 transmission electron microscope at 80 kv (three “reference” planes). Here, the landmarks used to identify the correct location in each section typically consisted of cross sections of small myelinated fibers and clusters of mitochondria transversing the section. Images were stored and analyzed by an observer blind to group assignment.

The observer counted all of the synapses in the hippocampal sections and the physical dissector method was again used to calculate the synapse density, for which the number of synapses present in the reference section and not the look-up section was counted (*Q*_synapse_).

The total number of synapses within an unbiased counting frame of a known area (*A*_frame_) was counted (1620 *μ*m^2^ per brain at 50,000x for the ultrathin sections). Again, the dissector volume of tissue (*V*_dis_) is calculated using the formula from above, where H is section thickness (70 nm) multiplied by the number of sections. Synapse density, *N*_vsynapse_, was calculated using the following formula: *N*_vsynapse_ = *Q*_synaps_/*V*_dis_ (using ultrathin sections).

### 2.6. Synaptic Ultrastructure

Synaptic ultrastructure, including thickness of postsynaptic density (thickest part), and synaptic curvature were measured by transmission electromicroscopic methods [10].

### 2.7. Statistical Analysis

Data were analyzed using SPSS software. One-way analysis of variance was used for synaptic density comparisons. *P* < 0.05 was considered statistically significant.

## 3. Results

Analysis using the dissector methods showed a significant increase in the mean synaptic number per *μ*m^3^ in hippocampus of morphine treated groups (1.85 synapse per *μ*m^3^) compared to placebo (0.8 synapse per *μ*m^3^) and control (0.67 synapse per *μ*m^3^) groups (*P* < 0.0001). It reflects a positive significant relationship between synaptic density increases and morphine consumption (*P* < 0.0001). There was not any significant difference between placebo and control groups (*P* < 0.585). Here, the 95% confidence intervals for morphine groups, placebo, and control groups were 2.0930–1.6069, (0.9293–0.6706), and (0.8209–0.5910), respectively (Table 1).

### 3.1. The Changes of Synaptic Curvature (SCZ) between the Three Groups (Morphine, Placebo, and Control Groups) (Table 2 and Figures 1 and 2)

As shown in Table 2, the analysis of hippocampal synaptic curvatures in rats that received morphine revealed a significant increase in the convex form of synapse (87.5%) compared to those of placebo (19.5%) and control groups (12.5%) (*P* < 0.0070). Here the concave form of synapses in morphine groups makes only 12.5% of all synapses compared to those of control (45%) and placebo groups (39%).

### 3.2. The Changes of Postsynaptic Thickness between Morphine, Placebo, and Control Groups

**Figure 1:**
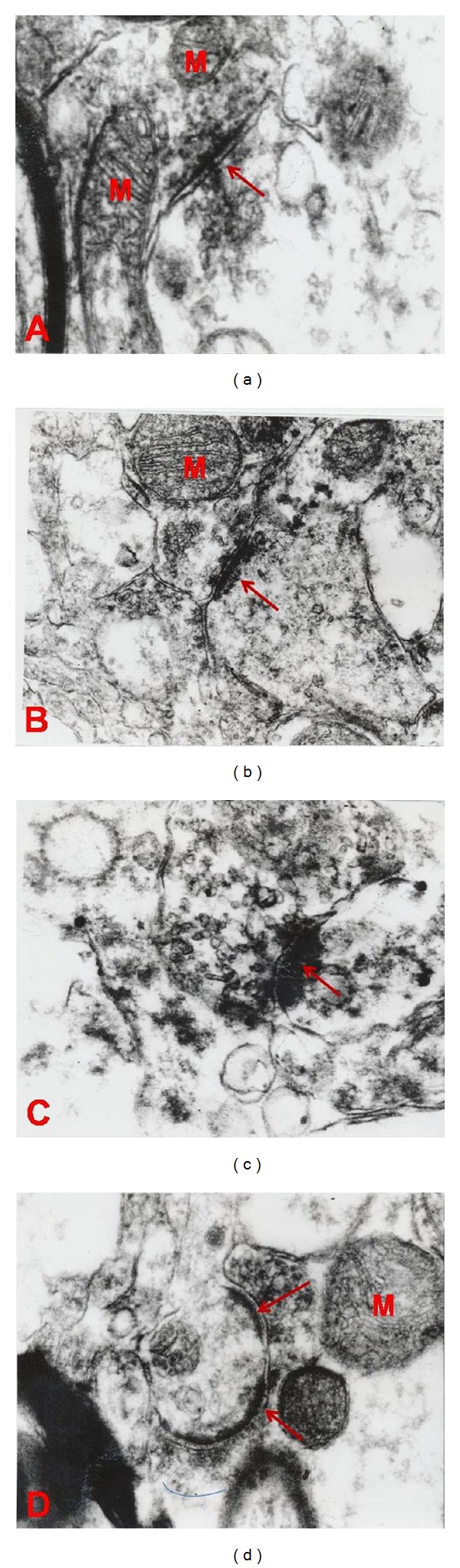
Electron microscopy of CA1 area of hippocampus: (a) control, (b) placebo group, and (c) and (d) morphine treated group (magnification: ×50000, scale bar: 0.5 *μ*m).

**Figure 2:**
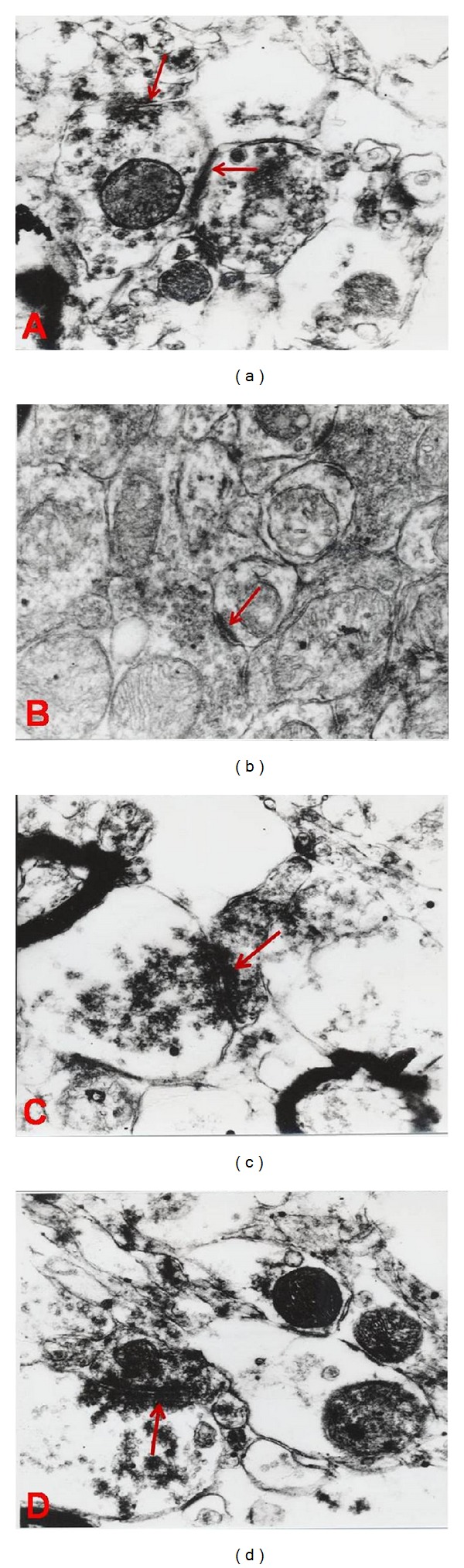
Electron microscopy of CA1 area of hippocampus: (a) control, (b) placebo group, and (c) and (d) morphine treated group (magnification: ×30000, scale bar: 0.5 *μ*m).

The statistical analysis also revealed a significant difference between groups in terms of thickness of postsynaptic density. As shown in the table below the minimum and maximum mean of thickness in morphine groups were 3 nm  and 5.7 nm, respectively (SD = 1.6 and *F* = 20.37). These results compared to those of placebo and control groups show significant increases in thicknesses of postsynaptic density (*P* < 0.0001). See Table 3 and Figures 1 and 2.

As shown in Figure 1, the synaptic contact zone curvature membrane is mostly convex in morphine groups (see the arrows in Figures 1, 2(c), and 2(d)) relative to control (see Figures 1 and 2(a)) and placebo groups (see Figures 1 and 2(b)). M indicates the mitochondria.

As shown in Figure 2, Postsynaptic density (PSD) is thick in morphine groups (see arrows in Figures 2(c) and 2(d)) relative to control (see Figure 2(a)) and placebo groups (see Figure 2(b)).

## 4. Discussion

The aims of our present study were to determine the chronic effects of morphine consumption on synaptic number, the synaptic curvature, and synaptic thickness of the rat hippocampus.

Analysis of our present data showed that chronic consumption of morphine has positive effects on the number of synapse in rat's hippocampus. In other words, the consumption of morphine for a long time causes a significant increase in the total number of synapses (synaptic density) in hippocampal CA1 area in rats. These results are in agreement with those of Kauer and Malenka in 2007, Kelley in 2004, and Berke and Hyman 2000 and Vorel et al. 2001 findings [10, 16, 20, 21].

They reported that synaptic plasticity is essential for neuroadaptations that resulted from a wide range of environmental stimuli. They also stated that use of the morphine, as environmental stimuli, for a long time can cause long-term changes in behavior by altering the synaptic structure, function, and number of synapses (the synaptic plasticity) in relevant brain circuits [10]. And also Alcantara et al. in 2011 and Pattij et al. in 2009 showed that morphine causes behavioral changes in animals. These morphine-induced behavioral changes are due to high sensitization of neuron to morphine consumption and changes in the number, function, and structure of synapse [4, 25].

It is thought that the structural and morphological changes of synapses, generation of new synapses, and synaptic plasticity have a main role in normal learning and memory process [14]. These findings suggested immediately that these processes are similar to associative learning and are essential in the early development of addiction.

In this study, an overall increase in the synaptic contact zone curvature was observed in the CA1 area of hippocampus of morphine-treated animals. According to Medvedev et al. in 2010 and Connor et al. in 2006, the synapses curvature was classified as follows: concave, a protrusion of presynaptic terminal into the postsynaptic button; convex, a protrusion of postsynaptic button into the pre-synaptic terminal; flat, no discernable curvature [26, 27].

The increase in synaptic contact zone curvature in our work was mostly convex, a protrusion of postsynaptic button into the pre-synaptic terminal, in morphine groups relative to placebo and control groups. This finding is in agreement with Agnihotri, Suzuki reports [27, 28]. According to them, the curvature of synaptic membrane changes equally during normal development either as convex or concave, but this change in curvature of synaptic membrane in morphine treated groups was mostly convex. These show that morphine dependence may cause unusual changes in cytoskeleton structure of postsynaptic contact zones of dendritic spines of neurons [29].

And also our data showed that the postsynaptic thicknesses change in morphine treated groups compared to other groups (placebo and control groups). It has been reported that the changes in postsynaptic density thicknesses have a close relationship to the alteration of protein profile, cytoskeletal, and morphology of postsynaptic site. According to the literatures, the postsynaptic density (PSD) refers to the concentration of protein components in postsynaptic site, including signaling, receptors, and scaffold protein; these proteins receive and transduce synaptic information [30, 31].

According to the reports, it was concluded the postsynaptic density plays an important role in synaptic regulation and plasticity [32–36]. It also has been reported that the levels of these proteins are strongly influenced by several environmental factors, such as opiate drugs. For example, the repeated administration of morphine induced significant changes in the levels of postsynaptic proteins [32, 36, 37] In one of the studies, Morón et al. 2007 [32] examined the levels of protein expression in synaptic membranes obtained from mouse hippocampus upon morphine administration using ICAT technology. They found that chronic morphine exposure induced an increase in the levels of proteins in postsynaptic site. Referring to the above results, the changes of synaptic curvature (SCZ) between the three groups (morphine, placebo, and control groups) it is likely has been shown that chronic consumption of opiates can significantly change the functions, structures, and morphology of neural systems, so it is likely that the increase of postsynaptic protein levels may cause a significant increase in postsynaptic density thicknesses as indicated by transmission electron microscopy in our experimental study. It is concluded that chronic morphine consumption plays a main role in the ultrastructural changes and synaptic plasticity in rats.

## Figures and Tables

**Table 1:** Changes in synaptic number in the morphine treated group.

Groups	Synaptic density per 1 × *μ*m^3^	95% confidence interval	*P* value
Morphine	1.85	2.0930–1.6069	*P* < 0.0001
Placebo (sucrose)	0.8	0.9293–0.6706	
Control	0.67	0.8209–0.5910	

**Table 2:** The changes of synaptic curvature (SCZ) between the three groups (morphine, placebo, and control groups).

Groups	Synaptic curvatures				
	*N*	Convex form	Concave form	Flat (no changes)	*P*
Morphine	8	87.5%	12.5%	0%	*P* < 0.0070
Placebo (sucrose)	8	19.5%	39%	41.5%	
Control	8	12.5%	45%	42.5%	

**Table 3:** Changes in thickness of postsynaptic density (nm) in the studied groups.

Groups	*N*	*X*	SD	SE	95% Confidence Interval	*F*	*P*
Morphine	8	4.37	1.6	0.565	3–5.7		
Placebo (Sucrose)	8	1.5	0.53	0.189	1–1.9	20.37	*P* < 0.001
Control	8	1.37	0.74	0.263	0.75–2		
