# Corrigendum to “Effect of Chronic Morphine Consumption on Synaptic Plasticity of Rat's Hippocampus: A Transmission Electron Microscopy Study”

**DOI:** 10.1155/2017/4153268

**Published:** 2017-07-31

**Authors:** Mohammad Hassan Heidari, Abdollah Amini, Zohreh Bahrami, Ali Shahriari, Abolfazl Movafagh, Reihane Heidari

**Affiliations:** ^1^Department of Biology and Anatomical Sciences, Faculty of Medicine, Shahid Beheshti University of Medical Sciences, Tehran, Iran; ^2^Department of Anesthesiology, Roozbeh Hospital, Tehran University of Medical Sciences, P.O. Box 1417653761, Tehran, Iran; ^3^Department of Medical Genetic, School of Medicine, Shahid Beheshti University of Medical Sciences, Tehran, Iran; ^4^Otorhinolaryngology Research Center, Tehran University of Medical Sciences, Tehran, Iran

In the article titled “Effect of Chronic Morphine Consumption on Synaptic Plasticity of Rat's Hippocampus: A Transmission Electron Microscopy Study” [1], the name and the affiliation of the fifth author were given incorrectly as “Abolfazle Movafag, Department of Genetic Science, Medical Faculty, Shahid Beheshti University of Medical Sciences, Tehran, Iran.” The revised authors' list and affiliations are shown above. Additionally, the email address of the corresponding author should be changed to “ashahriari@tums.ac.ir.”

## References

[1] Heidari M. H., Amini A., Bahrami Z., Shahriari A., Movafag A., Heidari R. (2013). Effect of chronic morphine consumption on synaptic plasticity of rat’s hippocampus: a transmission electron microscopy study. *Neurology Research International*.

